# Intestinal Barrier Dysfunction Exacerbates Neuroinflammation via the TLR4 Pathway in Mice With Heart Failure

**DOI:** 10.3389/fphys.2021.712338

**Published:** 2021-08-06

**Authors:** Jun-Yu Huo, Wan-Ying Jiang, Ting Yin, Hai Xu, Yi-Ting Lyu, Yuan-Yuan Chen, Meng Chen, Jie Geng, Zhi-Xin Jiang, Qi-Jun Shan

**Affiliations:** Department of Cardiology, the First Affiliated Hospital of Nanjing Medical University, Nanjing, China

**Keywords:** heart failure, neuroinflammation, intestinal barrier, lipopolysaccharide, TLR4

## Abstract

**Aims:**

The present study aimed to investigate alterations in neuroinflammation after heart failure (HF) and explore the potential mechanisms.

**Methods:**

Male wild-type (WT) and Toll-like receptor 4 (TLR4)-knockout (KO) mice were subjected to sham operation or ligation of the left anterior descending coronary artery to induce HF. 8 weeks later, cardiac functions were analyzed by echocardiography, and intestinal barrier functions were examined by measuring tight junction protein expression, intestinal permeability and plasma metabolite levels. Alterations in neuroinflammation in the brain were examined by measuring microglial activation, inflammatory cytokine levels and the proinflammatory signaling pathway. The intestinal barrier protector intestinal alkaline phosphatase (IAP) and intestinal homeostasis inhibitor L-phenylalanine (L-Phe) were used to examine the relationship between intestinal barrier dysfunction and neuroinflammation in mice with HF.

**Results:**

Eight weeks later, WT mice with HF displayed obvious increases in intestinal permeability and plasma lipopolysaccharide (LPS) levels, which were accompanied by elevated expression of TLR4 in the brain and enhanced neuroinflammation. Treatment with the intestinal barrier protector IAP significantly attenuated neuroinflammation after HF while effectively increasing plasma LPS levels. TLR4-KO mice showed significant improvements in HF-induced neuroinflammation, which was not markedly affected by intestinal barrier inhibitors or protectors.

**Conclusion:**

HF could induce intestinal barrier dysfunction and increase gut-to-blood translocation of LPS, which could further promote neuroinflammation through the TLR4 pathway.

## Introduction

In recent years, the link between the heart and brain in cardiovascular disease has received increasing attention. Previous studies have shown that cognitive impairment is highly prevalent in heart failure (HF) patients (Havakuk et al., 2017; Huynh et al., 2021). A more recent study further pointed out that the associated cognitive decline gradually developed after, but not before, cardiovascular disease (CVD) (Xie et al., 2019). However, there is still no effective treatment for this kind of CVD-related complication in the central nervous system (CNS), which not only affects patient prognosis but also greatly increases the social burden (Offer et al., 2019; Sierra, 2020). Recent studies have revealed that neuroinflammation is the early stage that precedes functional changes in the CNS (Chitnis and Weiner, 2017) and has been observed in both mice with HF and CVD patients (Thackeray et al., 2018). Thus, scientific studies to evaluate the pathogenesis underlying neuroinflammation after HF will highly valuable for CVD patients.

At present, the severity of abnormalities in the intestinal flora after CVD has been shown to gradually increase (Pasini et al., 2016; Jie et al., 2017; Tang et al., 2017). Evidence of the gut microbiome and intestinal dysfunction in CVD is accumulating. However, the change in intestinal barrier function may be underappreciated. Recently, Drapala et al. (2020) reported that rats with HF caused by spontaneous hypertension exhibited intestinal barrier dysfunction and increased intestinal permeability, which could drive intestinal microbiota translocation into systemic circulation, further activate the systemic immune response and exacerbate damage to other organs (Chen et al., 2019). In particular, the intestinal flora metabolite lipopolysaccharide (LPS) is closely associated with a variety of CNS diseases (Zhao et al., 2017). Studies have reported that increased levels of LPS can impair blood-brain barrier (BBB) function (Banks and Robinson, 2010) and are associated with activation of proinflammatory pathways in brain tissue (Kinsner et al., 2006). Therefore, damage to the intestinal barrier and the release of metabolites into the circulation after HF may be an important cause of neuroinflammation in CVD and might even be a vital target for the interaction between the heart and the brain.

Toll-like receptor 4 (TLR4) is an important pattern recognition receptor associated with inflammation that plays a crucial role in a variety of CNS diseases (Rahimifard et al., 2017). In brain tissue, TLR4 is mainly expressed in microglia (Buchanan et al., 2010). The activation of TLR4 and its downstream pathway could promote the activation of microglia and facilitate the release of inflammatory factors in the brain (Leitner et al., 2019). Furthermore, previous studies have shown that the intestinal flora metabolite LPS is a vital activator of TLR4 that can specifically binds to TLR4 and activates its downstream proinflammatory signaling pathway (Kinsner et al., 2006; Hamanaka et al., 2020). Accordingly, we hypothesize that HF could facilitate intestinal barrier dysfunction and increase the release of LPS, which could further activate TLR4 and promote neuroinflammation. Thus, in the present study, we first examined the changes in the intestinal barrier and neuroinflammation in mice with HF caused by ligation of the left anterior descending coronary artery (LAD), then analyzed their relationship by the intestinal barrier protector intestinal alkaline phosphatase (IAP) and intestinal homeostasis inhibitor L-phenylalanine (L-Phe), and further explored the underlying mechanism in TLR4-knockout mice.

## Materials and Methods

### Animals and Study Design

All experimental protocols in this study were approved by the Ethics Committee of Nanjing Medical University and conducted in accordance with the European Convention for the Protection of Vertebrate Animals used for Experimental and other Scientific Purposes. A total of 36 male wild-type (WT) C57BL/10 mice and 36 TLR4^–/–^ (TLR4-KO) C57BL/10 mice (all 6 weeks old) were obtained from GemPharmatech (Nanjing, China). All mice were housed in a specific pathogen-free room with free access to standard chow and water.

After 1 week of acclimation, both types of mice were randomly divided into four groups: the control group (sham LAD ligation, *n* = 6), the HF group (LAD ligation, *n* = 10), the HF+IAP group (LAD ligation+IAP, *n* = 10), and the HF+L-Phe group (LAD ligation+L-Phe, *n* = 10). LAD ligation was performed at week 0. After LAD ligation, IAP was administered in drinking water containing 120 units/mL IAP (Kaliannan et al., 2013), and L-Phe was administered by gavage at a dose of 150 mM L-Phe in saline (200 μL) twice per day (Campbell et al., 2010), while the other groups of mice received equal amounts of saline. At 8 weeks after LAD ligation, after analyzing cardiac function and intestinal permeability, the surviving mice were euthanized, and their blood, heart, intestine (similar regions) and brain were collected.

### HF Model

The HF model was established by permanent ligation of the LAD, as previously reported (Du et al., 2018). In brief, mice were anesthetized by an intraperitoneal (i.p.) injection with 1% sodium pentobarbital (50 mg/kg), endotracheally intubated and mechanically ventilated (the tidal volume was controlled at 1 ml with 120 times respiratory rate). The left thoracotomy surgery was performed at the fourth intercostal space. The anterior surface of the heart was then exposed and the anterior descending branch of the left main coronary artery is ligated using a 7-0 silk suture to occlude the vessel (below the tip of the normally positioned left auricle). After ligation, a local pale could be seen at the surface of heart. Subsequently, the thoracic cavity and skin were sutured, and mice were placed on an electric blanket to kept warm until regained consciousness. Then, the mice were administered penicillin (100,000 U/day) intramuscularly for 3 days to prevent infection. Control mice underwent thoracotomy surgery without ligation.

### Echocardiography

Echocardiography was performed at 8 weeks (before sacrifice) after LAD ligation. After the mice were anesthetized with isoflurane, cardiac function was evaluated using a Vevo2100 (VisualSonics, Toronto, Ontario, Canada) system. The left ventricular end-systolic diameter (LVDs), left ventricular end-diastolic diameter (LVDd), left ventricular anterior wall diastolic thickness (LVAWd), fractional shortening (FS), and ejection fraction (EF) of each mouse were recorded. The investigators were blinded to the groupings of the mice.

### Intestinal Permeability *in vivo*

Intestinal permeability was measured by a FITC-labeled dextran method before the mice were sacrificed (Dey et al., 2019). Briefly, after being fasted for 4 h, all mice were gavaged with the permeability tracer FITC-labeled dextran (4 kDa) (40 mg/100 g, Sigma-Aldrich, Missouri, United States). Blood sampled were collected after 3 h. The fluorescence intensity of each sample was measured by a fluorescence spectrophotometer (excitation, 490 nm; emission, 520 nm), and FITC-dextran concentrations were calculated from standard curves generated by serial dilution of FITC-dextran.

### Histological Analysis

The heart and the intestinal tissue sections were routinely stained with Masson’s trichrome, and the intestinal tissue sections were also routinely stained with hematoxylin-eosin (H&E). To analyze Masson’s staining, five fields from each sample were randomly selected, and the collagen volume fraction (CVF) was determined by Image-Pro Plus 6.0 software (National Institutes of Health, NIH).

### Immunohistochemistry

An anti-ionized calcium binding adaptor molecule-1 (Iba-1) antibody (Servicebio, Wuhan, China) was used to specifically recognize microglia in the cerebral cortex. All sections were examined using a microscope (Nikon, Tokyo, Japan), and at least five random fields (×400) per section were analyzed.

### Immunofluorescence Analysis

The distribution and expression levels of the tight junction proteins Occludin and ZO-1, as well as tyrosine hydroxylase (TH) in intestinal tissues were measured by immunofluorescence. In brief, intestinal cryostat sections (5 μm) were subjected to antigen retrieval in citrate buffer and blocked with normal goat sera. The sections were probed with anti-occludin (Abcam, Cambridge, United Kingdom), anti-ZO-1 (Abcam, Cambridge, United Kingdom) or anti-TH (Cell Signaling Technology, Denver, United States) and reacted with Alexa Fluor-conjugated secondary antibodies, followed by nuclear staining with 4′,6-diamidino-2-phenylindole (DAPI; Servicebio, Wuhan, China). To determine the distribution and levels of CD68, immunofluorescence analysis was performed on cerebral cortex sections using anti-CD68 (Cell Signaling Technology, Denver, United States) and appropriate Alexa Fluor-labeled secondary antibodies. The fluorescent signals were analyzed by a fluorescence microscope (Nikon, Tokyo, Japan).

### Enzyme-Linked Immunosorbent Assay (ELISA)

The collected blood samples were centrifuged to prepare plasma samples, and the fresh mouse brain tissues were homogenized and centrifuged. The levels of TNF-α in brain samples were analyzed by ELISA using specific kits (Thermo Fisher Scientific, Waltham, MA, United States). The levels of lipopolysaccharide (LPS) and D-lactate in the blood samples were analyzed by ELISA using specific kit for LPS (Cusabio, Wuhan, China) and D-lactate (Sigma, St. Louis, United States), respectively. Similarly, the plasma norepinephrine (NE) levels were also measured by a specific kit (Mlbio, Shanghai, China).

### Western Blotting

The levels of Occludin and ZO-1 in the intestine and TLR4 and MyD88 in the brain relative to β-actin were quantified by Western blotting. In brief, tissue samples were homogenized in lysis buffer (Servicebio, Wuhan, China) and centrifuged. After the protein concentrations were determined using the bicinchoninic acid method, the lysates (30 μg/lane) were separated by sodium dodecyl sulfate-polyacrylamide gel electrophoresis on 10% gels and transferred onto polyvinylidene fluoride membranes. The membranes were blocked with 5% nonfat dry milk in phosphate-buffered saline (PBS) containing Tween 20 and probed with anti-occludin, anti-ZO-1, anti-TLR4 and anti-MyD88 (Cell Signaling Technology, Denver, United States) at 4°C overnight. After being washed, the bound antibodies were detected with appropriate horseradish peroxidase (HRP)-conjugated secondary antibodies (Servicebio, Wuhan, China) and visualized with enhanced chemiluminescence.

### Real-Time PCR Quantification

Total RNA was extracted from the intestinal tissues using Trizol reagent (Invitrogen, Carlsbad, United States) and reverse transcribed into complementary DNA (cDNA) according to the instructions of a Reverse Transcription Kit (Takara, Dalian, China). Subsequently, quantitative real-time polymerase chain reaction (qRT-PCR) was conducted using SYBR green PCR Master Mix (Applied Biosystems, Foster, United States) on the ABI-7900 Real-Time PCR Detection System (Thermo Fisher Scientific, NY, United States). The targeted gene expression levels were normalized to that of mouse housekeeping gene, GAPDH. The primer sequences are listed in Supplementary Table 1.

### Statistical Analysis

Quantitative data are expressed as the mean ± SEM. SPSS 16.0 software (SPSS Inc., Chicago, IL, United States) was used for statistical analysis, and GraphPad Prism 5 software (GraphPad Prism, San Diego, United States) was used for graphing. The differences among groups were analyzed by one-way ANOVA followed by the Newman-Keuls test. *P* < 0.05 was considered statistically significant.

## Results

### Effectiveness of the HF Model

**FIGURE 1:**
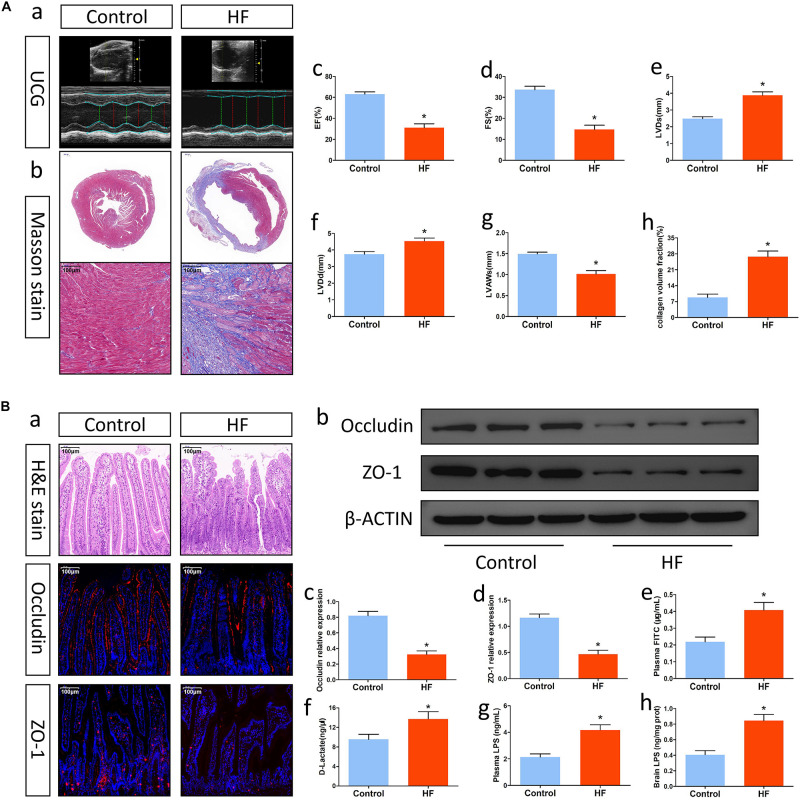
Alterations in cardiac function and intestinal barrier function after HF. **(A)** Changes in cardiac function. **(a)** Representative echocardiography tracings. **(b)** Representative images of Masson’s trichrome-stained cardiac tissue (magnification, 10× and 200×). **(c)** EF. **(d)** FS. **(e)** LVDs. **(f)** LVDd. **(g)** LVAWs. **(h)** Collagen volume fraction analysis of the results in **(b)**. **(B)** Alterations in intestinal barrier function after HF. **(a)** Representative images of H&E staining and representative immunofluorescent images of anti-occludin and anti-ZO-1 staining in the intestine (magnification, 200×). **(b)** Western blot analysis of the relative levels of Occludin, ZO-1 and expression of the control β-actin in intestinal tissue. **(c,d)** Quantitative analysis of Occludin and ZO-1 expression. **(e)** Plasma FITC-dextran concentrations in each group. **(f)** The levels of plasma D-Lactate in mice. **(g)** The levels of plasma LPS in mice. **(h)** The levels of brain LPS in mice. *P < 0.05 vs. the control group. EF, ejection fraction; FS, fractional shortening; LVDs, left ventricular end systolic diameter; LVDd, left ventricular end diastolic diameter; LVAWs, left ventricular anterior wall systolic thickness; LPS, lipopolysaccharide.

At 8 weeks after LAD ligation, there were 6 and 7 surviving mice in the control and HF groups, respectively. Cardiac function and pathological changes in each group were tested by echocardiography and Masson’s staining, respectively. Compared with mice in the control group, mice with HF showed increased LVDs and LVDd, reduced LVAWd, as well as decreased EF and FS (Figures 1Aa,c-g). Similarly, Masson’s staining also showed that mice with HF had obvious ventricular dilation and fibrous deposition (Figures 1Ab,h), suggesting that 8 weeks after LAD ligation, the HF model was successfully established.

### Mice With HF Showed Alterations in Intestinal Barrier Function

Heart failure might affect intestinal barrier function and promote gut injury. To understand the changes in intestinal barrier function after HF, we first examined pathological alterations and tight junction protein expression in gut tissue. H&E staining indicated that mice with HF had intestinal villus damage, reduced goblet cells and villus epithelial exfoliation (Figure 1Ba). Immunofluorescence analysis and Western blotting showed that intestinal expression of the tight junction proteins Occludin and ZO-1 in mice with HF was significantly lower than that in control mice (Figures 1Ba-d). Furthermore, analysis of FITC-labeled dextran and plasma D-lactate levels also indicated that intestinal permeability in the HF group was significantly higher than that in the control group (Figures 1Be,f). On this basis, we then explored the translocation of the microbiota metabolite LPS into systemic circulation. In comparison with mice in the control group, mice with HF showed significant increases in systemic LPS levels (Figure 1Bg,h). Accordingly, mice with HF had intestinal barrier dysfunction and increased gut-to-blood LPS translocation. Besides, the gut of HF mice also showed increased sympathetic activity, fibrosis and inflammation (Supplementary Figure 1).

### Mice With HF Had Enhanced Neuroinflammation, Which Was Associated With Intestinal Barrier Dysfunction

**FIGURE 2:**
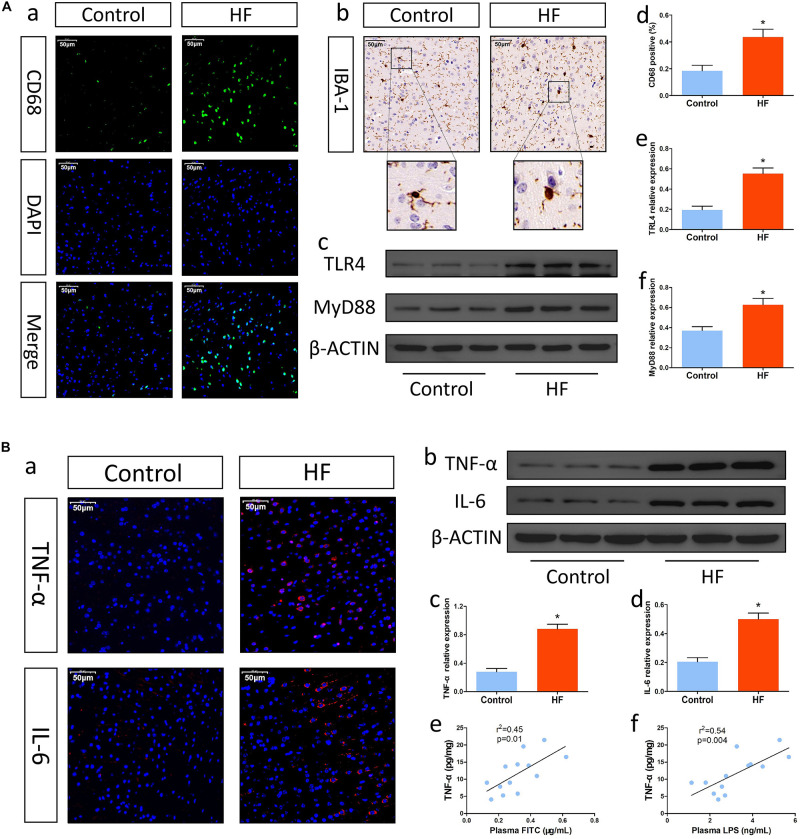
Changes in neuroinflammation after HF. **(A)** Changes in inflammatory cells and the proinflammatory pathway in brain tissue after HF. **(a)** Representative immunofluorescent images of anti-CD68 staining (magnification, 400×) in the brains of mice. **(b)** Representative immunohistochemical images of Iba-1 expression (magnification, 400×) in the brains of mice. **(c)** Western blot analysis of the relative levels of TLR4, MyD88 and the control β-actin in brain tissue. **(d)** Quantitative analysis of CD68-positive microglia in the brain. **(e,f)** Quantitative analysis of TLR4 and MyD88 expression. **(B)** Alterations in inflammatory cytokines in brain tissue after HF. **(a)** Immunofluorescence analysis of TNF-α and IL-6 expression (magnification, 400×) in the brains of mice. **(b)** Western blot analysis of the relative levels of TNF-α, IL-6 and the control β-actin in brain tissue. **(c,d)** Quantitative analysis of TNF-α and IL-6 expression. **(e)** Correlation analysis of brain TNF-α levels and plasma FITC-dextran concentrations. **(f)** Correlation analysis of brain TNF-α levels and plasma LPS levels. *P < 0.05 vs. the control group. Iba-1, ionized calcium binding adaptor molecule-1; TLR4, Toll-like receptor 4; MyD88, myeloid differentiation factor 88; LPS, lipopolysaccharide.

Intestinal injury is usually associated with neuroinflammation. Thus, we first measured the expression of the proinflammatory pathway factors TLR4 and MyD88, which are important for microglial activation. According to the Western blot results, mice in the HF group showed relatively higher levels of TLR4 and MyD88 than control mice (Figures 2Ac,e,f). Then, we examined the activation of inflammatory microglia. As shown in Figures 2Aa,d, the frequency of CD68-positive microglia in the HF group was higher than that in the control group. Similarly, immunohistochemistry also revealed that microglia in the brain cortex in the HF group had hypertrophied cytoplasms and retracted processes, while microglia in the control group had small cytoplasms and thin processes (Figure 2Ab), indicating that mice with HF had obvious microglial activation. Next, we investigated the levels of inflammatory cytokines. Immunofluorescence analysis and Western blotting showed that the levels of TNF-α and IL-6 were significantly increased in the brains of mice with HF in comparison with those of control mice (Figures 2Ba-d). Furthermore, the levels of inflammatory cytokines in the brain were positively correlated with intestinal permeability and plasma LPS levels to some extent (Figures 2Be,f). Collectively, mice with HF had increased neuroinflammation, which might be associated with intestinal barrier dysfunction.

### Intestinal Barrier Protectors Attenuated Neuroinflammation in Mice With HF

**FIGURE 3:**
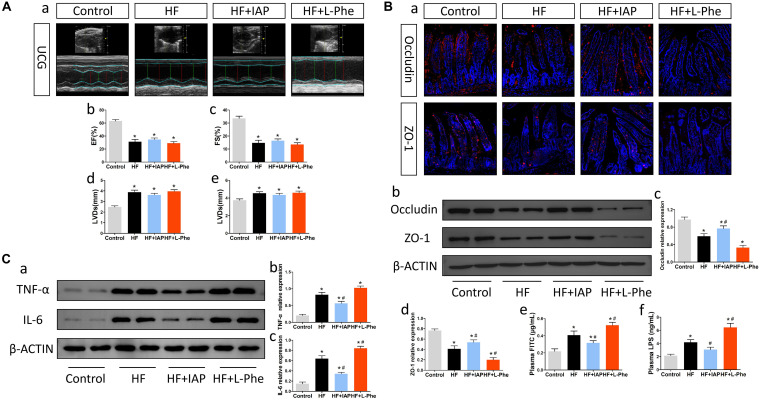
IAP attenuated neuroinflammation in mice with HF. **(A)** Cardiac function in each group. **(a)** Representative echocardiography tracings. **(b)** EF. **(c)** FS. **(d)** LVDs. **(e)** LVDd. **(B)** Intestinal barrier function in each group. **(a)** Representative immunofluorescent images of anti-occludin and anti-ZO-1 staining in the intestine (magnification, 200×). **(b)** Western blot analysis of the relative levels of Occludin, ZO-1 and the control β-actin in intestinal tissue. **(c,d)** Quantitative analysis of Occludin and ZO-1 expression. **(e)** Plasma FITC-dextran concentrations in each group. **(f)** The levels of plasma LPS in each group. **(C)** Levels of brain inflammatory cytokines in each group. **(a)** Western blot analysis of the relative levels of TNF-α, IL-6 and the control β-actin in brain tissue. **(b,c)** Quantitative analysis of TNF-α and IL-6 expression. *P < 0.05 vs. the control group; ^#^*P* < 0.05 vs. the HF group. EF, ejection fraction; FS, fractional shortening; LVDs, left ventricular end systolic diameter; LVDd, left ventricular end diastolic diameter; LPS, lipopolysaccharide; IAP, intestinal alkaline phosphatase; L-Phe, L-phenylalanine.

To understand the relationship between intestinal barrier dysfunction and neuroinflammation in mice, we investigated whether the intestinal barrier protector IAP could improve neuroinflammation after HF. IAP is a kind of tissue-specific alkaline phosphatase, which specifically plays a protective role in intestinal tissue and could effectively protect the intestinal barrier (Lallès, 2014). After oral administration of IAP, mice with HF showed significant improvements in gut tight junction expression, intestinal permeability and plasma LPS levels (Figure 3B). Although the intestinal sympathetic activity, fibrosis and inflammation (Supplementary Figure 1), as well as cardiac dysfunction (Figure 3A) was not significantly attenuated, the expression of inflammatory cytokines in the brain was effectively moderated by IAP treatment (Figure 3C). Additionally, treatment with L-Phe, which inhibits endogenous IAP activity and injures intestinal homeostasis (Ghosh and Fishman, 1966; Koyama et al., 2002), exacerbated HF-related neuroinflammation to some extent (Figure 3C) while increasing intestinal permeability and plasma LPS levels (Figure 3B). Therefore, these data indicated that injury to the intestinal barrier and increased gut-to-blood microbiota metabolite translocation after HF might be underlying causes that promote the progression of HF-related neuroinflammation.

### TLR4-KO Mice Exhibited Improvements in Neuroinflammation After HF and Were Not Obviously Affected by Intestinal Barrier Inhibitors or Protectors

**FIGURE 4:**
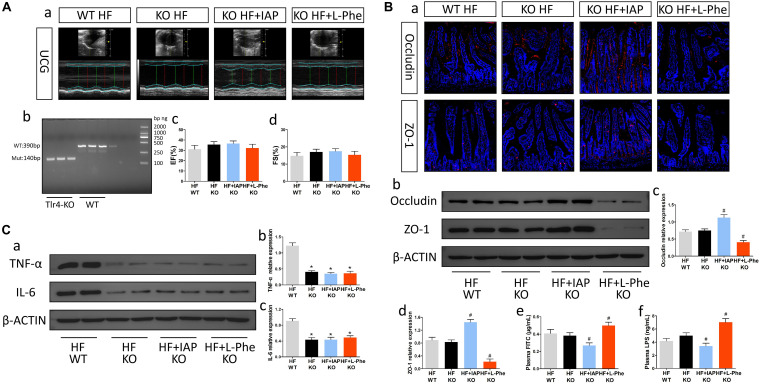
TLR4-KO mice exhibited improvements in neuroinflammation after HF and were not obviously affected by intestinal barrier inhibitors or protectors. **(A)** Cardiac function in each group. **(a)** Representative echocardiography tracings. **(b)** Verification of TLR4 knockout. **(c)** EF. **(d)** FS. **(B)** Intestinal barrier function in each group. **(a)** Representative immunofluorescent images of anti-occludin and anti-ZO-1 staining in the intestine (magnification, 200×). **(b)** Western blot analysis of the relative levels of Occludin, ZO-1 and the control β-actin in intestinal tissue. **(c,d)** Quantitative analysis of Occludin and ZO-1 expression. **(e)** Plasma FITC-dextran concentrations in each group. **(f)** The levels of plasma LPS in each group. **(C)** Levels of brain inflammatory cytokines in each group. **(a)** Western blot analysis of the relative levels of TNF-α, IL-6 and the control β-actin in brain tissue. **(b,c)** Quantitative analysis of TNF-α and IL-6 expression. *P < 0.05 vs. the WT HF group; ^#^*P* < 0.05 vs. the KO HF group. EF, ejection fraction; FS, fractional shortening; LPS, lipopolysaccharide; IAP, intestinal alkaline phosphatase; L-Phe, L-phenylalanine.

Previous studies have shown that the gut microbiota metabolite LPS might be an important ligand for TLR4. To explore the potential mechanism by which intestinal injury promotes neuroinflammation after HF, we constructed TLR4-KO mice and induced HF (Figure 4A). Compared with WT mice, TLR4-KO mice showed significant decreases in TNF-α and IL-6 in brain tissue after HF (Figure 4C). Furthermore, the protective effect did not obviously affected after alteration of LPS level by IAP or L-Phe treatment (Figures 4B,C). Thus, after HF, intestinal barrier dysfunction and metabolite entry into circulation may promote neuroinflammation through the TLR4 pathway.

## Discussion

In the present study, we used a mouse HF model to evaluate the changes in LPS levels and neuroinflammation and further explored the potential mechanism of HF-related neuroinflammation in TLR4-KO mice. We observed that (Huynh et al., 2021) mice with HF showed obvious increases in intestinal permeability and plasma LPS levels, which were accompanied by elevated expression of TLR4 in the brain and enhanced neuroinflammation (Havakuk et al., 2017) the intestinal barrier protector IAP attenuated neuroinflammation after HF while effectively increasing plasma LPS levels; and (Xie et al., 2019) TLR4-KO mice showed significant improvements in HF-induced neuroinflammation, which were not markedly affected by intestinal barrier inhibitors or protectors.

### HF and Intestinal Barrier Dysfunction

At present, interactions between organs have raised much attention. Intestinal abnormalities caused by HF are gradually being discovered. Previous basic studies revealed that HF animal models exhibited obvious gut dysfunction and intestinal dysbacteriosis (Carrillo-Salinas et al., 2020; Seo et al., 2020). Consistently, Kummen et al. (2018) also found that HF patients had decreased microbial richness and altered gut microbiota through profiling 2 independent cohorts. However, there have been few reports on HF-related intestinal barrier damage. After HF, decreased cardiac output and function can induce digestive tract hypoperfusion and gastrointestinal ischemia and edema, which can promote damage to the intestinal tight junctions and gut mechanical barrier (Nagatomo and Tang, 2015). In addition, overactivation of the sympathetic nervous system after HF may also impair intestinal function, further exacerbating intestinal permeability (Santisteban et al., 2017). Recently, Zhou et al. (2018) reported intestinal barrier dysfunction and increased permeability in rats with myocardial infarction (MI) and patients with ST-segment elevation. Similarly, in the present study, we observed decreased expression of the intestinal tight junction proteins Occludin and ZO-1 in mice with HF, indicating injury to the intestinal barrier. Moreover, we also found increased gut-to-blood translocation of FITC-dextran, the intestinal flora metabolite LPS and D-lactate, hinting at alterations in intestinal permeability after MI. Therefore, our data support the notion that HF can alter gut barrier permeability and induce microbiota metabolites into systemic circulation, which might further promote pathological changes in remote organs after HF.

### Intestinal Abnormality and CNS Disease

It has become evident that the intestinal microbiota and the gut-brain axis might play important roles in the development of CNS disease. Under physiological conditions, a healthy intestinal state and balanced microbial composition can promote the production of neuromodulators to maintain CNS function (Sharon et al., 2016). However, under pathological conditions, alterations in gut function and the microbiota contribute to neuroinflammation and can exacerbate CNS diseases such as Alzheimer’s disease (AD) and depression (Lach et al., 2018). Recently, Wang et al. (2019) reported that intestinal disorders are an important cause of neuropathology and cognitive impairment in AD mice. Similarly, in the cardiovascular field, related research also revealed that HF-related intestinal abnormalities are closely associated with CNS damage after HF (Yu et al., 2020). Our present study further showed that the intestinal flora metabolite LPS may serve as an important factor connecting gut alterations and neuroinflammation in mice with HF. Previous study has shown that intraperitoneal injection of LPS can directly induce microglial activation and neuroinflammation in the brain in rats (Ji et al., 2020). In the present study, we similarly observed that increases in intestinal permeability and plasma LPS levels were consistent with enhanced neuroinflammation in mice with HF, and the level of plasma LPS was positively correlated with inflammatory cytokine expression in the brain. In addition, we also found that intestinal barrier protectors that decreased plasma LPS levels could effectively improve neuroinflammation in mice with HF. Therefore, the increased gut-to-blood LPS translocation caused by HF-related intestinal barrier dysfunction might be an important cause and therapeutic target for neuroinflammation after HF.

### TLR4 and Neuroinflammation

Toll-like receptor 4 is a crucial pattern recognition receptor involved in inflammatory diseases (Akira et al., 2006). Rosa et al. (2021) recently reported that the TLR4 pathway promoted the activation of inflammatory cells in the brain and further exacerbated CNS injuries. Similarly, Tu et al. (2014) also showed that activation of TLR4 was associated with the CNS inflammatory reaction and brain damage, indicating the important role of TLR4 in neuroinflammation. Interestingly, intestinal flora metabolite LPS has recently been recognized as one of the specific ligands that activates TLR4. An in vitro study showed that LPS could directly activate TLR4 to promote microglial activation (Hamanaka et al., 2020). Likewise, an in vivo study also revealed that circulating LPS activated neuroinflammation through the TLR4 pathway and promoted the progression of cognitive dysfunction in mice (Zhang et al., 2018). Consistent with previous studies, in our present study, we similarly found that elevated LPS levels after HF increased TLR4 expression in brain tissue and enhanced neuroinflammation. Furthermore, TLR4-KO mice showed significant improvements in HF-induced neuroinflammation, and these effects were not markedly affected by increased LPS levels, while WT mice showed consistent alterations in neuroinflammation and LPS levels induced by intestinal barrier inhibitors or protectors. Accordingly, activation of the TLR4 pathway might be an important underlying mechanism of neuroinflammation caused by increased LPS levels after HF and might play a key role in HF-related intestinal injury and neuroinflammation.

In conclusion, HF could induce intestinal barrier dysfunction and increase gut-to-blood LPS translocation, which could further promote neuroinflammation through the TLR4 pathway.

### Limitations

First, the cognitive function of mice was not examined in this study. However, the relationship between neuroinflammation and cognitive impairment has been widely examined in both basic and clinical studies, indicating that neuroinflammation can promote the development of cognitive impairment (Okello et al., 2009; Kummer et al., 2015). Second, the endpoint of the present study was 8 weeks after LAD ligation. The alterations and mechanisms of acute neuroinflammation in the early stage after HF remain to be clarified. Furthermore, further studies are still required to illustrate the mobilization of immune cells and the signaling mechanisms underlying HF-related intestinal barrier dysfunction.

## Conclusion

Our present study indicated that HF induced intestinal barrier dysfunction and increase gut-to-blood LPS translocation, which could further promote neuroinflammation through the TLR4 pathway. Our findings might provide new therapeutic strategy for HF-related CNS disease.

## Data Availability Statement

The original contributions presented in the study are included in the article/Supplementary Material, further inquiries can be directed to the corresponding author/s.

## Ethics Statement

The animal study was reviewed and approved by all experimental protocols in this study were approved by the Ethics Committee of Nanjing Medical University.

## Author Contributions

Z-XJ and Q-JS designed the present study. J-YH and W-YJ made substantial contributions to animal model, histological analysis, and statistical analysis and wrote the initial draft of the manuscript. TY and HX made contributions to immunohistochemistry and immunofluorescence analysis. Y-TL made contributions to Western blotting. MC made contributions to ELISA. Y-YC and JG made contributions to echocardiography. Z-XJ revised it critically for the important intellectual content. Q-JS has given final approval of the version to be published. All authors have participated sufficiently in the work to take public responsibility for appropriate portions of the content and approved the manuscript, as well as agreed to be accountable for all aspects of the work, and read and approved the final manuscript.

## Conflict of Interest

The authors declare that the research was conducted in the absence of any commercial or financial relationships that could be construed as a potential conflict of interest.

## Publisher’s Note

All claims expressed in this article are solely those of the authors and do not necessarily represent those of their affiliated organizations, or those of the publisher, the editors and the reviewers. Any product that may be evaluated in this article, or claim that may be made by its manufacturer, is not guaranteed or endorsed by the publisher.

## Supplementary Material

Supplementary Figure 1 Alterations in gut sympathetic activity, fibrosis and inflammation of mice. **(a)** Representative images of anti-TH immunofluorescent staining (magnification, 400×) and Masson staining (magnification, 400×) in the intestinal tissues of mice. **(b)** The levels of plasma NE in mice. **(c)** Quantitative analysis of anti-TH fluorescent signals in the intestine of mice. **(d)** CVF of the intestinal tissues of mice. **(e)** CD3 gene expression in the intestinal tissues of mice. **(f)** CD68 gene expression in the intestinal tissues of mice. **(g)** TLR2 gene expression in the intestinal tissues of mice. **(h)** RAGE gene expression in the intestinal tissues of mice. ^∗^*P* < 0.05 vs. the control group; ^#^*P* < 0.05 vs. the HF group. TH, tyrosine hydroxylase; NE, norepinephrine; CVF, collagen volume fraction; TLR2, toll-like receptor 2; RAGE, receptor for advanced glycation end-products.

Supplementary Table 1 Sequences of specific primer pairs.
