# Supplementary figures and images for: Intestinal Barrier Dysfunction Exacerbates Neuroinflammation via the TLR4 Pathway in Mice With Heart Failure
Source: Front Physiol. 2021 Aug 6;12:712338. doi: 10.3389/fphys.2021.712338 (PMC8378453; doi:10.3389/fphys.2021.712338)

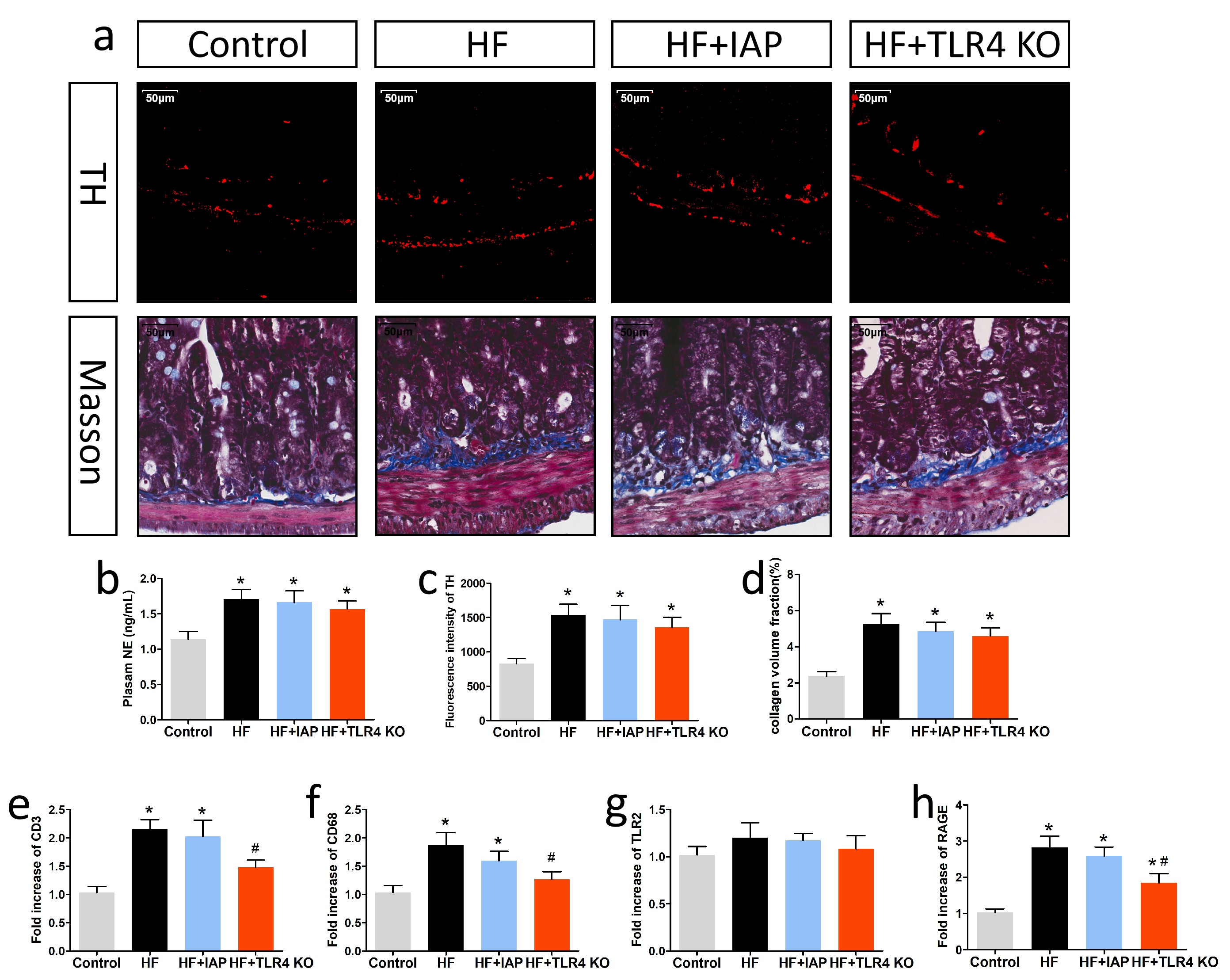

Supplement: Supplementary Figure 1 — Alterations in gut sympathetic activity, fibrosis and inflammation of mice. (a) Representative images of anti-TH immunofluorescent staining (magnification, 400×) and Masson staining (magnification, 400×) in the intestinal tissues of mice. (b) The levels of plasma NE in mice. (c) Quantitative analysis of anti-TH fluorescent signals in the intestine of mice. (d) CVF of the intestinal tissues of mice. (e) CD3 gene expression in the intestinal tissues of mice. (f) CD68 gene expression in the intestinal tissues of mice. (g) TLR2 gene expression in the intestinal tissues of mice. (h) RAGE gene expression in the intestinal tissues of mice. ∗P < 0.05 vs. the control group; #P < 0.05 vs. the HF group. TH, tyrosine hydroxylase; NE, norepinephrine; CVF, collagen volume fraction; TLR2, toll-like receptor 2; RAGE, receptor for advanced glycation end-products. [file Image_1.JPEG]
