# Supplementary material for: Intestinal Barrier Dysfunction Exacerbates Neuroinflammation via the TLR4 Pathway in Mice With Heart Failure
Source: Front Physiol. 2021 Aug 6;12:712338. doi: 10.3389/fphys.2021.712338 (PMC8378453; doi:10.3389/fphys.2021.712338)
Supplement: Supplementary Table 1 — Sequences of specific primer pairs. [file Table_1.DOCX]

**Supplementary table**

**Table S1. Sequences of specific primer pairs.**

| Primers | Sequence (5'→3') |
| --- | --- |
| CD68-Mus | TGTCTGATCTTGCTAGGACCG |
|  | GAGAGTAACGGCCTTTTTGTGA |
| CD3-Mus | GGTCCTGCCCCATTTATAG |
|  | GCCTTTTGCATTAGCAGAG |
| TLR2-Mus | CTCTTCAGCAAACGCTGTTCT |
|  | GGCGTCTCCCTCTATTGTATTG |
| AGER-Mus | CTTGCTCTATGGGGAGCTGTA |
|  | CATCGACAATTCCAGTGGCTG |
